# Effect of platelet mediator concentrate (PMC) on Achilles tenocytes: an in vitro study

**DOI:** 10.1186/s12891-016-1160-2

**Published:** 2016-07-22

**Authors:** Esra Arslan, Thomas Nellesen, Andreas Bayer, Andreas Prescher, Sebastian Lippross, Sven Nebelung, Holger Jahr, Christine Jaeger, Wolf Dietrich Huebner, Horst Fischer, Marcus Stoffel, Mehdi Shakibaei, Thomas Pufe, Mersedeh Tohidnezhad

**Affiliations:** Department of Anatomy and Cell Biology, RWTH Aachen University, Wendlingweg 2, D-52074 Aachen, Germany; Department of Prosthodontics and Biomaterials Centre of Implantology, Medical Faculty, University Hospital of RWTH Aachen University, Aachen, Germany; Department of Heart and Vascular Surgery, University Hospital of Schleswig-Holstein Campus Kiel, Kiel, Germany; Department of Molecular and Cellular Anatomy, RWTH Aachen University, Aachen, Germany; Department of Trauma Surgery, University Medical Center Schleswig-Holstein Kiel Campus, Kiel, Germany; Department of Orthopaedic Surgery, University Hospital of RWTH Aachen University, Aachen, Germany; Curasan AG, Kleinostheim, Germany; Department of Dental Materials and Biomaterials Research, University Hospital of RWTH Aachen University, Aachen, Germany; Institute of General Mechanics, RWTH Aachen University, Aachen, Germany; Institute of Anatomy Ludwig- Maximilians University Munich, Munich, Germany

**Keywords:** Tenocyte, Platelet, Platelet mediator concentrate, Scleraxis, Tenomodulin

## Abstract

**Background:**

Although there are many studies discussing the etiological and pathological factors leading to both, acute and chronic tendon injuries, the pathophysiology of tendon injuries is still not clearly understood. Although most lesions are uncomplicated, treatment is long and unsatisfactory due to the poor vascularity of tendon tissue. Platelet mediator concentrate (PMC) contains many growth factors derived from platelets, which can promote wound healing. In this study we investigate the effects of PMC on tenocyte proliferation and differentiation in order to provide an experimental basis for tissue regeneration strategies and to develop new treatment concepts.

**Methods:**

Using enzyme linked immunosorbent assay (ELISA) we were able to quantify the several growth factors and cytokines found in PMC. Tenocytes were isolated both from human and from mouse Achilles tendons and stimulated with PMC. CyQuant® and Cell Titer Blue® assays were carried out to analyze tendon growth and viability at different concentrations of PMC. Real time RT-PCR was used to analyze tenocyte gene expression with or without PMC treatment. Immunohistochemistry was carried out to detect the tenocyte-specific antibody tenomodulin (TNMD) and scleraxis (SCX).

**Results:**

We were able to detect numerous mediators such as platelet derived growth factor BB (PDGF-BB), interleukin 6 (IL-6), vascular endothelial growth factor (VEGF), tumor necrosis factor (TNF-α), transforming growth factor beta 1 (TGF-ß1), and bone morphogenetic proteins 2, 4 and 7 (BMP-4, BMP-2, BMP-7) in PMC. It was possible to show a positive effect of PMC on human tendon cell growth and viability in a dose-dependent manner. Furthermore, PMC treatment led to induction of gene expression of scleraxis (SCX), type I collagen A 1 (Col1A1) and TNMD by tenocytes.

**Conclusions:**

We suggest that the use of autologous PMC may be a suitable addition to conventional tendon therapy that is capable of increasing and optimizing tendon healing and reducing the risk of recurrence.

## Background

Between three and five million people worldwide suffer tendon and ligament injuries every year [1]. Achilles tendinopathy is often related to specific sports injuries caused by inappropriate biomechanical stress. It can also occur through non-usage due to sedentary lifestyles. Furthermore the occurrence in association with metabolic disorders such as arthritis and diabetes, or from the use of medications such as corticosteroids or certain antibiotics, underscores the multifactorial etiology of tendinopathy [2].

Tendon ruptures are classified as either spontaneous, traumatic, or open laceration. Previous histological examinations of spontaneously ruptured Achilles tendons have elucidated degenerative changes in a majority of cases [3]. Degenerative changes in the Achilles tendon emerge primarily in regions with hypo- or avascularity, such as at the insertion or tendon-waist [4]. Multiple microtrauma lead to high expression of VEGF, which is characteristic of the painful pathogenesis of tendon ruptures [5]. VEGF-mediated neoangiogenesis is found in areas with neovessels and accompanying nerves together with synthesis of matrix metalloproteinases (MMP) [6].

VEGF is not the only molecule to regulate tendon development and healing. Others include SCX, PDGF-BB and TGF-ß1 [7]. In general, growth factors play an important role in the several phases of tendon healing. There is an inflammatory, a proliferative, and a remodeling phase [7].

Tendon ruptures are currently managed either conservatively or surgically [8]. Regardless of treatment method, however, there is no guarantee that full function can be regained, and a recurrence risk after surgical treatment persists (1.7–5.4 %) [9, 10]. The ability of tendons to self-repair is limited. Treatment is thus long and unsatisfactory, and only 70 % of patients regain full previous tendon function [10]. Repaired tendon characterized by scarring, and its biomechanical properties are altered and inferior [11].

New approaches to treatment employ alternative strategies such as stem cell repair, platelet rich plasma (PRP) or other orthobiologics, either through direct application or in conjunction with scaffolding [1, 12, 13]. Studies indicate that autologous growth factors, in the form of PRP, have an accelerated curative effect especially in treating ruptured Achilles tendons [14–16]. PRP delivers local autologous bioactive agents which influence the tendon healing phases, inflammation, extracellular matrix (ECM) synthesis and angiogenesis [17]. Platelet-released growth factors (PRGF) have a similar effect. This is a concentrated form of platelets without both erythrocytes and leukocytes [16].

However, there is no standardized method of plasma preparation. Randomized clinical trials are few [13]. The Advanced Tissue Regeneration Kit (ATR®) manufactured by Curasan AG Kleinostheim offers a standardized, labor-free approach. It delivers a centrifugation-free method of preparing human platelet mediator concentrates (PMC) [18]. The kid can be used chairside in the visiting room and is ready in a few hours. The present study detects different mediators in PMC and compares then with plasma and serum. Furthermore the effects of PMC on human and mouse tenocytes are elucidated in vitro.

## Methods

Substantial growth factors were detected and quantified in PMC. The levels of these growth factors in PMC were compared to those of plasma and serum. The PMC were used as stimuli to treat tenocytes.

### PMC preparation

The ATR® kit is a sterilized, disposable medical product. PMC were prepared as described in the guide and other work by Schmolz et al. [18]. First 8 ml whole peripheral blood from healthy, mainly male donors between the ages of 20–35 was drawn into a 10 ml syringe. There it was mixed with a combination of an anticoagulant and a sedimentation accelerator (Solutions A and B). This blood mixture rests for approximately 50 to 60 min in an upright position as provided for by the kit, at room temperature. The plasma supernatant containing the platelets was then transferred into a 5 ml syringe. 3.5 ml supernatant was then injected into the top opening of the ATR system, where the platelets pass through different filter systems. Solution C, which contains a washing buffer, was then added. It eliminated any anticoagulants or sedimentation fragment. Solution D was the last to be injected. It has triggerd the release of mediators from the platelets. The injection proceed in two steps. First 1 ml of solution D was added and incubated at room temperature for 10 min. Then the rest of the solution was pressed onto the filter. The triple-concentrated PMC, containing only the mediators, was collected at the lateral opening of the kit using another syringe. We aliquoted the PMC in 200 μL Eppis and stored it at −80 °C.

### Serum and plasma preparation

Blood from 14 human volunteers (18–45 years, 5 male, 9 female) was collected, from which whole blood (10 ml) was kept to clot at room temperature for approximately 10 h and centrifuged at 2,000 × g for 10 min at 37 °C to the serum. Plasma was prepared from whole blood from same volunteers in a second syringe containing anti-coagulant (citrate) and centrifuged in the same way. Each serum and plasma sample was stored in aliquots at −20 °C until further use (i.e., ELISA analyses).

### Isolation of human tenocytes

Tenocytes were isolated from the Achilles tendon of postmortem human donors under written declarations of consent obtained from each donor enrolled in this study; these are on record at the Department of Anatomy. The tendon was washed with phosphate buffered saline (PBS) and cut into pieces after careful removal of the epitendineum. To remove the fibroblasts, the tissues were pretreated with 0.5 % trypsin for 5 min at 37 °C. Subsequently, the tendon pieces were washed and cultivated in Dulbecco’s modified Eagle’s medium (DMEM) (GIBCO®, Invitrogen) containing 50 % fetal calf serum (FCS) and 1 % penicillin streptomycin Fungizone (PSF) [19]. After approximately two weeks, tenocytes continuously migrated from this explant and adhered to petri dishes. After 70–80 % confluence, cells were removed using 1 % trypsin (5×; Gibco, Invitrogen) and cultivated in culture medium (DMEM) (GIBCO®, Invitrogen) containing 100 unit/ml penicillin, 100 μg/ml streptomycin (GIBCO®, Invitrogen) and 10 % FCS. The tenocytes were used from the second through fourth passages.

### Isolation of mouse tenocytes

Tenocytes were isolated from the Achilles tendons of mice between two and three days after birth (balb/mouse; charles river). First, the skin and epitendineum were carefully removed from the Achilles tendon. Tissue was segmented and treated with 1 % trypsin (5×; Gibco, Invitrogen) for five minutes at 37 °C. After washing with PBS, the tendon was cultivated in culture medium containing 50 % FCS [20]. The concentration of the FCS was reduced to 10 % FCS after one week. After two weeks cultivation, a 70–80 % confluence had been achieved. Passaging was then performed with 1 % trypsin in PBS.

### Stimulation of cells with PCM

After the second passage, the adherent monolayer was trypsinized and seeded in Petri dishes at 10^5^ cells/cm^2^ cells, and then cultivated in culture medium. One day prior to stimulation, the concentration medium was replaced by serum-starved medium containing 1 % FCS. Stimulation with PMC ensued at concentrations of 0, 1, 5 and 10 % in FCS starved medium for 3 h and 24 h.

### Cell proliferation and cell viability assay

The CyQuant® assay Kit (Invitrogen GmbH, Darmstadt, Germany) was employed to measure in vitro tenocyte cell proliferation according to the manufacturer’s protocol. The CellTiter-Blue® Cell viability assay (Promega Corporation, Madison, USA) was used to evaluate cell viability in response to various concentrations of PCM. Human tenocytes treated according to the stimulation protocol above were used for both assays. The results were normalized to control group without PMC addition.

### Analysis of mediator composition of PMC

The concentrations of different mediators like PDGF-BB, VEGF, TGF-β1, BMP-2, BMP-4, BMP-7, TNF-α and IL-6 were assessed in PCM, serum and plasma using commercially available ELISA kits (R&D Systems and Peprotech).

### Real time RT-PCR

RNA was extracted with NucleoSpin RNA XS (Machery Nagel, Germany) according to the manufacturer’s protocol. The RNA concentration was determined by photometric analysis using the NanoDrop 1000 system (PEQLAB Biotechnologie GmbH). 1 μg of total RNA was then digested with DNase I (Roche GmbH) and transcribed into cDNA by reverse transcription with RevertAidTM reverse transcriptase (Fermentas Life Sciences). Real time PCRs were processed in triplicate using the ABI StepOnePlusTM apparatus (Applied Biosystems) in a total volume of 15 μl containing 70–100 ng of cDNA, gene specific primers and SYBR Green I reagent (Applied Biosystems). The target genes tenomodulin (TNMD), scleraxis (SCX) and Collagen1A1 (Col1A1) (Qiagen, Germantown, MD, USA) were measured. Beta-2-microglobulin (B2M) served as internal control.

### Immunofluorescence

For immunofluorescence, tenocytes were seeded and cultivated on cover slides. Having been washed thrice with PBS, cells were fixed in Zamboni for 10 min and washed again with PBS. Cells were stored until use in PBS at 4 °C. The cells were then washed thrice with 0.1 % Triton X in PBS for 5–10 min and then thrice with TRIS for 5 min. Afterwards cells were blocked with 1.5 % bovine serum albumin (BSA) in TRIS for 20 min. Immunostaining against tenomodulin (Santa Cruz Biotechnology, CA, USA; sc-49352) (1:50 in Tris-buffered saline, 2 h) and scleraxis (Santa Cruz Biotechnology, CA, USA; sc-49352) (1:50 in Tris-buffered saline, 2 h) was used to characterize the differentiated phenotype of the tenocytes. Phalloidin Alexa Fluor 488 (life technologies, A12379, 1:20) and Alexa Fluor 594 (life technologies, A 21431, 1: 350) (1:200 in 1.5 % BSA in TRIS, 2 h) was used subsequently. A bisBenzimide staining was used to counterstain the nuclei. To analyze the expression of SCX and TNMD in tenocytes, three (3) images from each sample were captured randomly. Images were made using a Keyence BZ-9000 microscope with a 20× PlanFlour El NA 0.45 Ph1 objective and equal exposure time. Expression of SCX and TNMD were determined using the freely available software FIJI [21]. Contoures of tained cells were followed with the freeform tool to define regions of interest (ROI-1) and their mean grey value was determined. In parallel, the mean grey value of the cell-near background was determined (ROI-2) and substracted from ROI-1 to calculate the mean overall value per picture. The values were normalized to the control group.

### Statistical analysis

Results were expressed as the mean ± standard error (SEM) and compared using a one-way ANOVA, Dunnett’s post hoc test. To compare the growth factor content of PMC, plasma and serum, we used a one-way ANOVA, Bonferroni test. Differences were considered significant at *p* ≤ 0.05. All statistical graphs and analyses were created with GraphPad Prism 5.0 (GraphPad Software, La Jolla, CA, USA).

## Results

**Fig. 1:**
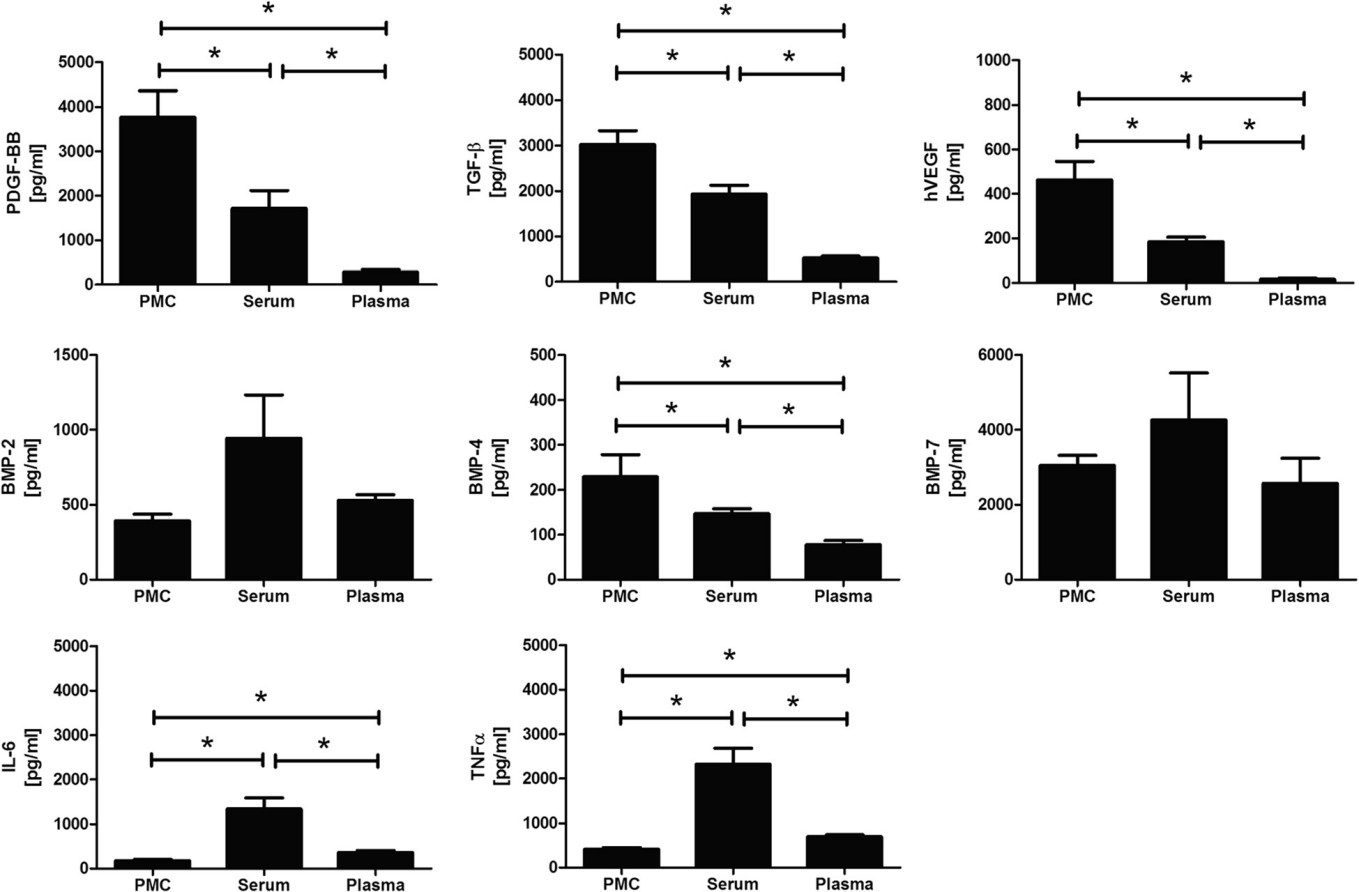
Concentrations of growth factors in PMC, serum and plasma. In PMC and serum the PDGF-BB concentration was substantially elevated compared to plasma (3767 ± 593 pg/ml and 1715 ± 399 pg/ml compared to 283 ± 55.8 pg/ml, *n* = 7, *p* < 0.05 ). The concentrations of VEGF, TGF-ß and BMP-4 were likewise increased in PMC and serum (VEGF: 464 ± 82.3 pg/ml and 185 ± 20.5 pg/ml to 185 ± 20.5 pg/ml, *n* = 14; TGF-β: 3022 ± 304 pg/ml and 1936 ± 192 pg/ml to 515 ± 45.8, *n* = 7; BMP-4: 230 ± 48.9 pg/ml and 147 ± 11.7 pg/ml to 77.7 ± 9.58, *n* = 9, respectively, *p* < 0.05). No significant differences were measured between BMP-2 and BMP-7 levels in PMC, serum, or plasma (BMP-2: 77.7 ± 9.58 pg/ml, 943 ± 290 pg/ml and 943 ± 290; respectively; BMP-7: 3059 ± 272 pg/ml, 3059 ± 272 pg/ml and 2577 ± 670 pg/ml; respectively, *n* = 6, *p* < 0.05). The levels of both inflammatory cytokine IL-6 and TNF-α were significantly increased in serum as compared to PMC and plasma (IL-6: 1340 ± 242 pg/ml to 177 ± 28.3 pg/ml and 365 ± 64.4 pg/ml, respectively; TNF-α: 2321 ± 362 pg/ml to 413 ± 37.9 pg/ml and 695 ± 44.1 pg/ml, respectively, *n* = 6, *p* < 0.05)

Various mediators were detected using ELISA in PMC and compared with serum and plasma (Fig. 1). In PMC the PDGF-BB, hVEGF, TGF- β1 and BMP-4 concentrations were substantially elevated as compared to serum und plasma. At the same time the IL-6 and TNF-α levels were significantly lower than in serum and plasma. Data showed no significant difference in BMP-2 and BMP-7 levels of PMC as compared to plasma and serum.

**Fig. 2:**
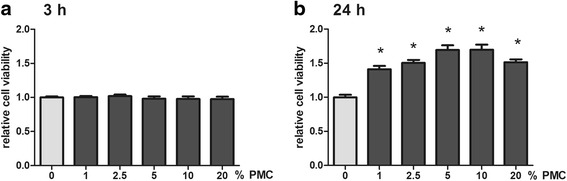
Cell viability. The cell viability rate was improved by application of PMC to the media. Cell viability was measured using a CTB assay at 3 h (**a**) and 24 h (**b**). No effect could be discerned after 3 h; but every concentration of PMC tested led to increased cell viability after 24 h

Cell viability was determined by using a CellTiter-Blue® Cell viability assay (Fig. 2). The cell viability rate improved with application of PMC as a media supplement at 3 h and 24 h. There was no effect after 3 h, but any concentration of PMC tested led to increased cell viability after 24. PMC increased the cell viability of tenocytes positively, in a concentration dependent manner, after 24 h (B) of stimulation (1.0 ± 0.036, 1.41 ± 0.049, 1.50 ± 0.044, 1.69 ± 0.069, 1.70 ± 0.076 and 1.51 ± 0.043 by 0, 1, 2.5, 5, 10 and 20 % PCM, *n* = 6, *p* ≤ 0.05); but no effect appeared after 6 h treatment (A).

**Fig. 3:**
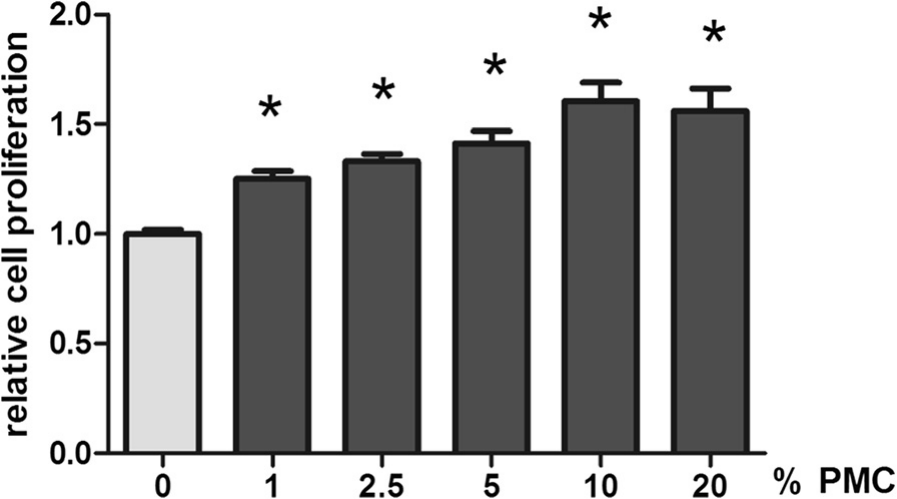
Cell proliferation. Tenocyte proliferation increased with PMC concentration. The cell proliferation assay was measured using CyQuant cell proliferation assay at 24 h

The proliferative effect of PMC on human Achilles tenocytes was tested in a CyQuant assay (Fig. 3). PMC concentrations of 0, 1, 2.5, 5, 10 and 20 % were tested. Significantly increased cell proliferation was observed in tenocytes at each PMC concentrations used (1.00 ± 0.21, 1.25 ± 0.035, 1.33 ± 0.034, 1.41 ± 0.057, 1.61 ± 0.085 and 1.56 ± 0.10 by 0, 1, 2.5, 5, 10 and 20 % PCM, 24 h, *n* = 14, *p* ≤ 0.05).

**Fig. 4:**
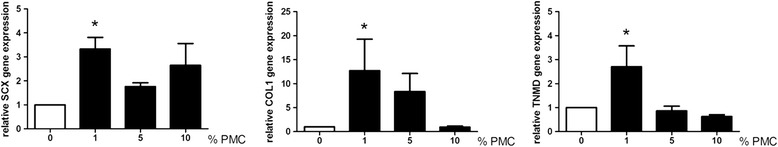
Gene expression of SCX, COL1 and TNMD after PMC treatment. Only the addition of 1 % PMC to the tenocyte culture medium showed significantly increased gene expression of the tenocyte specific markers SCX, COL1 and TNMD as compared to 5 and 10 % PMC supplementation (SCX: 3.33 ± 0.48 compared to 1.76 ± 0.16 and 2.65 ± 0.90, respectively *n* = 5; COL1: 12.7 ± 6.55 compared to 8.37 ± 3.75 and 0.94 ± 0.22, respectively, *n* ≥ 6 , TNMD: 2.7 ± 0.87 compared to 0.86 ± 0.19 and 0.63 ± 0.08, respectively, *n* ≥ 4, *p* ≤ 0.05)

To assess whether PMC influences the tendon-specific gene expression, we used real time PCR. The expression of SCX, COL1 and TNMD genes was determined after treatment with different concentrations of PMC (Fig. 4). Gene expression analysis confirmed a significant induction of these genes only at 1 % PMC (3.33 ± 0.48, 12.7 ± 6.55 and 2.4 ± 0.74 respectively, *n* ≥ 5, *p* ≤ 0.05).

**Fig. 5:**
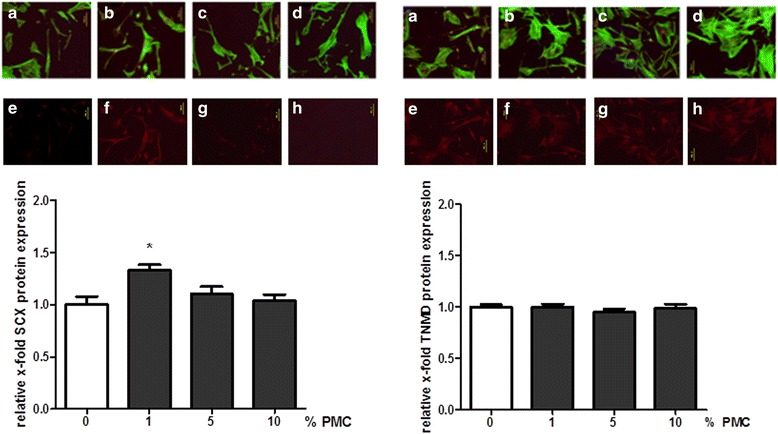
Protein expression of SCX and TNMD after PMC treatment. Protein expressions of SCX (*red*) and TNMD (*red*) were detected using immunofluorescence. Phalloidin staing (*green*) displayes the cell morphology. The addition of 1 % PMC to the tenocyte culture medium (**b**, **f**) showed significantly increased SCX protein expression in tenocytes. There was no effect on TNMD protein expression. Fluorescence intensity was measured and quantified using the software Image J. (**a** and **e**: 0 %, **b** and **f**: 1 %, **c** and **g**: 5 %, **d** and **h**: 10% PMC)

In order to confirm these results, immunofluorescence was used as a means of assessing the effect of PMC on tenocytes. This analysis revealed an increased relative expression of SCX in tenocytes upon stimulation with 1 % PMC. (The relative x-fold SCX protein expression was 1.33 ± 0.54 for 1 % PMC, 1.10 ± 0.07 for 5 % PMC and 1.04 ± 0.06 for 10 % PMC versus 1.0 ± 0.08 for the control group, *n* ≥ 5, *p* ≤ 0.05.) There was no significant change in TNMD expression after PMC treatment (1.0 ± 0.03 for 1 % PMC, 0.95 ± 0.03 for 5 % PMC and 0.98 ± 0.04 for 10 % PMC versus 1.0 ± 0.02 for the control group; *n* ≥ 5, *p* ≤ 0.05) (Fig. 5).

## Discussion

Many clinical and laboratory results have been published that show positive healing effects of orthobiologics like PRP, stem cells, PRGF, platelet rich fibrin (PRF), and other platelet compositions in tendon repair [22, 23]. Nevertheless, the different methods of preparing various platelet compositions do not guarantee the same biological response. This prevents a suitable comparison of results [24] [25]. Because the ATR® kit employs a standardized method to deliver a mediator composition, its use allows for mutual comparison of results. Such a standardized method of preparing PMC could provide an alternative means of achieving similar biological effects in patients, particularly in a clinical setting.

The results described above demonstrate that PMC contains most of the important mediators in tendon healing: PDGF-BB, hVEGF, TGF-ß1, BMP-4, BMP-2 and BMP-7. The concentration of the mediators was considerably higher than that found in the plasma and serum. PDGF-BB was increased by a factor of 13, hVEGF by a factor of 24, and TGF-ß1 and BMP-4 each by a factor of 3.

Prior studies had already shown that PDGF-BB exerts an accelerating effect by stimulating the migration and proliferation of tenocytes and progenitor cells [14]. Recent in vitro studies have also demonstrated the bacteriostatic effect of TGF-ß1 and PDGF-BB in blood components like PRP [26]. Additionally, TGF-ß1 contributes to the formation of scars as well as fibronectin expression in tendon healing, which is less positive [27, 28]. VEGF, by contrast, appears to have a somewhat harmful effect on in vitro tendon healing as it induces angiogenesis and matrix metalloproteinases (MMPs). It thus downgrades the material properties of the Achilles tendon [6]. However, in chronic wounds characterized by insufficient blood supply, angiogenesis is an important factor to improve healing, particularly during the early phase of tendon healing [29].

This study quantified a low level of TNF-α and IL-6 in PMC. Prior studies have addressed the importance of various anti-inflammatory factors during the early phase of tendon healing. Most recently Ackermann et al. demonstrated an up-regulation of the anti-inflammatory cytokines IL-6, IL-8 and Il-10 as well as a resolution of the inflammatory phase two weeks after Achilles tendon rupture in humans [30]. Andersen et al. suggest that IL-6 is involved in the transformation of mechanical loading into collagen synthesis [31]. TNF-α is upregulated in tendon lesions and has been found to be an important regulating factor in that context. It stimulates additional production of the pro-inflammatory cytokines interleukin 1 beta (IL-1ß), TNF-α, and anti-inflammatory cytokin IL-10 , whereas its expression is reduced in loaded as compared to unloaded tendon repair [11, 32]. Furthermore TNF-α leads to suppression of type 1 collagen and to extracellular matrix (ECM) degradation through stimulation of MMPs. ECM causes damage and scar formation during tendon healing. Taken together, pro-inflammatory cytokines seem to have both positive and negative effects on tendon healing. As PMC only contains lesser amounts of TNF-α, we suggest that it acts as less of a trigger of inflammation after application.

PMC also contains variants BMPs, which have been described as inducing adipogenic, osteogenic and chondrogenic differentiation of mesenchymal stem cells (MSCs) and tendon-derived stem cells (TDSCs) in vitro [33]. The addition of PMC to human Achilles tenocytes leads to a dose-dependent significant enhancement of tenocyte proliferation and viability. Thus we can exclude a toxic response.

Tendon tissues harbor a unique cell population, termed tendon stem/progenitor cells (TSPCs), which has universal stem cell characteristics such as self-renewal capacity. The effect of PMC on TSPCs is not clear [34].

Another major advantage of PMC is that it only involves mediators without foreign antigens (*e.g*., blood group antigens, HLA molecules) such as may cause an immunogenic responses [18]. The great importance of this is shown in an unpublished paper within our task group proving the apoptotic effect of platelet poor plasma (PPP) on cells, where the addition of a minimal quantity of PRP eliminated the condition. Using real time RT-PCR we were able to quantify the expression of the tendon-typical genes TNMD, SCX and Col1A1 following treatment of mouse tenocytes with PMC. Tendons are naturaly a largely avascularized tissue and under physiological condition a high amount of blood-derived growth factors may not be required. We could show that lower concentrations of PMC are more effective in inducing tenogenic marker gene expression than higher amounts PMC amounts. We hypotheize that a high amount of growth factors released from platelets are more suitable for regeneration of high vascularized tissues such as bone [35] and skin. Schweitzer et al. described SCX as a differentiation-associated transcription factor expressed in progenitors and tendon tissue cells. It was found to be an important factor in early tendon formation [36, 37]. Col1A1 is important for the stability of tendon tissue. With immunohistochemistry we were able to assess the effect of PMC on tenocytes. The treatment of tenocytes with 1 % PMC leads to increased SXC expression. There was no significant difference in TNMD expression after either treatment or non-treatment of tenocytes with PMC. TNMD is seen as an important regulator of tenocyte proliferation and involvement in collagen fibril maturation [38].

The distribution of types I and III collagen is critical for the tensil strength of tendons [39]. We could demonstrate that a low amount of PMC leads to increased typ I collagen synthesis in monolyer culture. The mechanical properties of tendons cannot be determined in monolayer cultivation. Furthermore, tenocytes in monolayer culture display an unstable phenotype and tend to de-differentiate [40]. Additional experiments in three-dimensional cultures are nessecary to analyse effects of PMC on phenotype differentiation and function of these cells, as well as the organization of the synthetized collagen network.

It is possible to identify tenocytes in vitro with immunohistochemistry; in culture mediums of higher passages, tenocytes display a tendency toward phenotypic drift and dedifferentiation [41–43].

We were able to demonstrate that PMC can be an additional source of the already various expression of endogenous growth factors and cytokines in tendon healing.

## Conclusions

As previously discussed, between three and five million people worldwide are afflicted with tendon and ligament injuries every year. Achilles tendinopathy is often related to specific sport injuries caused by inappropriate biomechanical stress. Here we provide evidence that PMC exerts a biological effect on tenocytes by promoting tenocyte growth and by upregulating tenogen specific markers. We hypothesize that low concentrations of PMC can accelerate the tendon healing and reduce both scar formation as well as the typically high risk of recurrence upon tendon loading. We intend to explore this question by investigating the effect of platelet mediators on tenocytes under non-physiological strain conditions in a bioreactor. Furthermore, long-term in vitro studies should investigate the effect of PMC treatment on ECM components. In vivo studies will be necessary in order to confirm the results.

## Abbreviations

BMP, bone morphogenetic protein; BSA, bovine serum albumin; C, grad celsius; COL1A1, type I collagen A 1; DMEM, Dulbecco’s modified Eagle’s medium; ELISA, enzyme linked immunosorbent assay; FCS, fetal calf serum; G, gravitational; h, hour; H, human; IF, immunofluorescence; IL, interleukin; min, minute; MMP, matrix metalloproteinase; MSC, mesenchymal stem cells; PBS, phosphate buffered saline; PDGF-BB, platelet derived growth factor BB; PMC, platelet mediator concentrate; PPP, platelet poor plasma; PRP, platelet rich plasma; PSF, penicillin streptomycin Fungizone; SCX, scleraxis; SEM, standard error; TDSC, tendon derived stem cell; TGF-ß, transforming growth factor beta; TNF, tumor necrosis factor; TNMD, tenomodulin; VEGF, vascular endothelial growth factor
